# Fe(Se,Te) Thin Films Deposited through Pulsed Laser Ablation from Spark Plasma Sintered Targets

**DOI:** 10.3390/ma17112594

**Published:** 2024-05-28

**Authors:** Michela Iebole, Valeria Braccini, Cristina Bernini, Andrea Malagoli, Nicola Manca, Alberto Martinelli, Matteo Cialone, Marina Putti, Shiv J. Singh, Giovanna Latronico, Paolo Mele

**Affiliations:** 1 Shibaura Institute of Technology, Omiya Campus, 307 Fukasaku, Minuma-ku, Saitama City 337-8570, Japan; michela.iebole@spin.cnr.it (M.I.); i048025@shibaura-it.ac.jp (G.L.); pmele@shibaura-it.ac.jp (P.M.); 2 Physics Department, University of Genova, Via Dodecaneso 33, 16146 Genova, Italy; matteo.cialone@unige.it (M.C.);; 3 CNR—SPIN Genova, Corso Perrone 24, 16152 Genova, Italy; cristina.bernini@spin.cnr.it (C.B.); andrea.malagoli@spin.cnr.it (A.M.); nicola.manca@spin.cnr.it (N.M.); alberto.martinelli@spin.cnr.it (A.M.); 4 RAISE Ecosystem, 16152 Genova, Italy; 5 Institute of High-Pressure Physics (IHPP), Polish Academy of Sciences, Sokołowska 29/37, 01-142 Warsaw, Poland; shivjees@gmail.com; 6 International Research Fellow of Japan Society for the Promotion of Science (JSPS), 5 Chome-3-1 Kojimachi, Chiyoda City, Tokyo 102-0083, Japan

**Keywords:** iron-based superconductors, thin films, pulsed laser ablation, spark plasma synthesis, critical current density

## Abstract

Iron-based superconductors are under study for their potential for high-field applications due to their excellent superconducting properties such as low structural anisotropy, large upper critical fields and low field dependence of the critical current density. Between them, Fe(Se,Te) is simple to be synthesized and can be fabricated as a coated conductor through laser ablation on simple metallic templates. In order to make all the steps simple and fast, we have applied the spark plasma sintering technique to synthesize bulk Fe(Se,Te) to obtain quite dense polycrystals in a very short time. The resulting polycrystals are very well connected and show excellent superconducting properties, with a critical temperature onset of about 16 K. In addition, when used as targets for pulsed laser ablation, good thin films are obtained with a critical current density above 10^5^ A cm^−2^ up to 16 T.

## 1. Introduction

Iron-based superconductors (IBSs) discovered in 2008 [1] have sparked great interest thanks to their relatively high critical temperatures T_c_, their very high critical fields H_c2_ and their critical current densities J_c_, which match those required for applications in different sectors [2,3,4,5]. Among the many IBS families, compounds belonging to the so-called 11-system are very interesting: despite their relatively low critical temperature (around 15 K for bulk Fe(Se,Te) [2], which reach up to 21 K in thin films due to compressive strain [6]), they exhibit lower toxicity if compared to other iron-based superconductors containing arsenic [7], easy phase synthesis and high and nearly isotropic upper critical fields.

For practical applications, superconductors need to be manufactured as wires or tapes. Powder-in-tube is the technique most commonly used for fabricating long lengths of conductors, being easily scalable and cost effective. The manufacturing of Fe(Se,Te) wires through the powder-in-tube technique is not a viable option, since no sheath has been found yet which can provide good performance [8]. On the other hand, it has been observed that Fe(Se,Te) has excellent properties when grown on the technical substrates designed and commercialized for high-temperature superconductor REBCO (Rare Earth-Barium-Copper Oxide) materials: the so-called coated conductors (CCs) [9,10]. This technology is based on the deposition of superconducting films on metallic biaxially textured templates covered by a series of suitable buffer layers [11]; however, its potential applications are severely limited by its cost, which is ultimately related to the high degree of complexity of the manufacturing process. Fe(Se,Te) CCs are attractive because they could significantly reduce the substrate complexity: Fe(Se,Te) requires simpler deposition conditions than REBCO (a deposition temperature between 200 °C and 400 °C in an ultrahigh vacuum instead of above 800 °C in oxygen) and has a greater J_c_ tolerance to grain-to-grain misalignment (about 9° versus 3–4° for REBCO) [12]. The template structure can thus be simplified from the complex multi-layer buffer architecture required in REBCO CCs, which promotes a strict epitaxial growth and prevents substrate ion diffusion, to the single layer required for Fe(Se,Te) [2,4,13,14,15,16].

In order to simplify the template structure for Fe(Se,Te) CCs, a single buffer layer of Zr-doped CeO_2_ (CZO) as thin as 25 nm grown by metal–organic decomposition (MOD) on Yttria-stabilized zirconia (YSZ) and on NiW substrates has been successfully used to obtain high-quality superconducting Fe(Se,Te) films [2,17,18], proving that a simplified architecture Ni-W/CZO-(MOD)/Fe(Se,Te) is suitable for the design and fabrication of long-length iron-based CCs.

Another issue to be faced in order to obtain high-quality thin films is the standard of the target. In particular, in order to obtain homogeneous and highly reproducible films, with no particulates on the film, a high-density polycrystal is required, which deteriorates slowly with the laser ablation [19]. Very long solid-state reactions and/or melting with subsequent annealing [20,21] are needed for this purpose and they can take up to several days. To achieve faster sintering processes, the spark plasma sintering (SPS) method has been applied, e.g., to achieve fast densification of K-doped Ba122 (this is in contrast to the conventional synthesis method, which requires several tens of hours); after only 5 min of SPS, optimally K-doped bulks with near theoretical density were obtained [22]. This technique has the advantage of being not only very fast but also easily scalable to achieve large targets in a short time [23].

In this paper, we describe the preparation and the characterization of Fe(Se,Te) polycrystals prepared through SPS. We then proceed to the deposition of a Fe(Se,Te) thin film on a CaF_2_ substrate in order to check the quality of the SPS target for the PLD purpose. We analyze the growth, the superconducting properties and the pinning mechanisms of thin films deposited through PLD, starting from Fe(Se,Te) SPS targets.

## 2. Materials and Methods

High-purity FeSe_0.5_Te_0.5_ powders were synthesized by the solid-state reaction method. Stoichiometric amounts of Fe and Te powders (99.9% purity) and Se powders (99.999% purity) supplied by Alfa Aesar were well mixed through manual grinding and blending in an agate mortar following the FeSe_0.5_Te_0.5_ stoichiometry in a glovebox under Ar atmosphere, then sealed under vacuum in borosilicate-glass tube, and finally heated up to 500 °C for 100 h [21]. The result is a homogeneous black powder. For SPS, the FeSe_0.5_Te_0.5_ powders were ground to an average size of 100 microns in order to homogenize the particles and facilitate their densification into a pellet. These powders were placed in a graphite die with an internal diameter of 15 mm, which was closed by two graphite punches. The pellet was sintered in the die using a SPS LABOX-210 (NJS Co., Ltd., Yokohama, Japan) Sinterland under a constant pressure of 45 MPa in vacuum. The sample was heated from room temperature to 680 °C in 8 min and to 700 °C in 3 min. After 15 min, it was allowed to cool down to room temperature before releasing the pressure. The pellet was removed from the graphite dies and then polished to remove traces of the protective graphite foil. The resulting pellet was 5 mm thick and 15 mm in diameter; however, through this technique, pellets up to 10 cm in diameter can be easily fabricated.

The sample was tested and used as a target to grow Fe(Se,Te) thin films by pulsed laser deposition (PLD) on (001)-oriented CaF_2_ single-crystal substrates. The deposition was carried out in a high-vacuum PLD system equipped with a Nd:YAG laser at 1024 nm; the optimized laser parameters to obtain high-quality epitaxial thin films were a 3 Hz repetition rate, a 2 J cm^−2^ laser fluency (2 mm^2^ spot size) and a 5 cm distance between the target and the sample. The deposition was carried out at a pressure of 10^–8^ mbar while the substrate was kept at 300 °C. The resulting Fe(Se,Te) thin films are typically about 100 nm thick [16].

The microstructure of the pellet was investigated using a Leica Cambridge S360 scanning electron microscope equipped with an Oxford X-Max 20 energy dispersive spectrometer (EDS) to assess the grain size, the chemical homogeneity and the stoichiometry of the phase. Both the pellet and the thin films were analyzed by X-ray diffraction for phase identification and to assess their crystalline orientation. The experiment was carried out in a Bragg–Brentano configuration using a Cu anticathode. In order to define the critical temperature T_c_, we measured the magnetic susceptibility of a slab-shaped sample 6 mm long and 3 mm wide, with a thickness of about nine hundred microns that was cut from the pellet. The measurement was performed with the magnetic field applied along the slab plane in a 5.5 T Magnetic Properties Measurement System by Quantum Design equipped with a dc-superconducting quantum interference device (SQUID) magnetometer.

The thin film was patterned with Hall bars of 20 × 50 μm^2^ geometry in order to perform transport properties on a defined geometry. Patterning was carried out by standard UV lithography followed by water-cooled argon ion milling (Ar energy was 500 eV). After the milling process, the sample was cleaned by mild sonication in acetone for a few tens of seconds and dried in nitrogen flow [24]. Transport measurements were carried out in a 16 T cryogen-free superconducting magnet by cryogenic treatment, with a room temperature bore with diameter of 85 mm and a variable temperature insert (VTI) for measurements in the range 1.6 K–325 K. Resistivity measurements as a function of the applied magnetic field were performed either on the target and on the thin film by the standard four-probe measurement technique. Critical current values at different temperatures and magnetic fields were extracted from voltage versus current characteristics acquired by sweeping the current from zero with exponentially increasing steps with the field parallel and perpendicular to the film surface.

## 3. Results

### 3.1. Pellet

**Figure 1:**
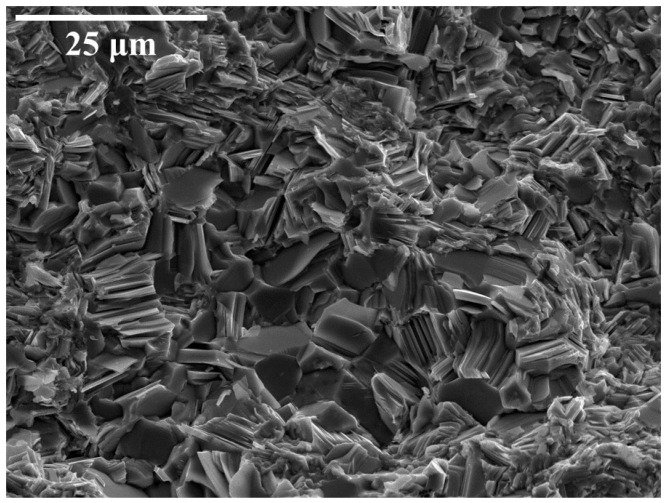
Secondary electron image of the fractured pellet prepared by SPS.

**Figure 2:**
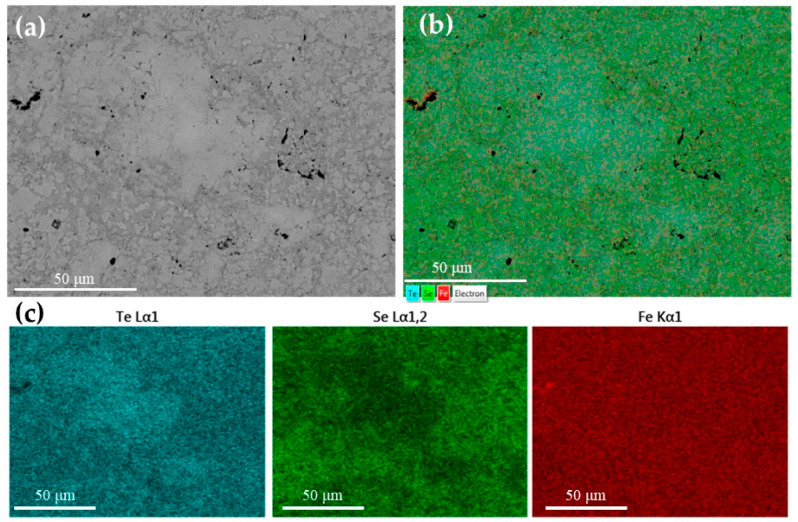
(**a**) Back-scattered SEM image, (**b**) layered image and (**c**) elemental maps of the pellet prepared by SPS performed using EDS.

Figure 1 shows the secondary electron (SE) image of the fractured pellet: the sample is constituted of a dense cluster of micrometric crystals characterized by a tabular shape. Despite the global stoichiometry measured through SEM being exactly FeSe_0.5_Te_0.5_, elemental mapping performed using EDS revealed a significative fluctuation of the Se/Te ratio in different regions of the sample. From these maps, shown in Figure 2, we observe the presence of regions richer in tellurium in the larger grains and others richer in selenium occurring in the smaller grains. While iron (in red) is uniformly present, we observe that tellurium-rich regions (light blue) alternate with selenium-rich ones (green). This inhomogeneity in the Se/Te ratio is commonly observed in bulk Fe(Se_1−x_Te_x_) and is related to a thermodynamic instability of the Fe(Se_0.5_Te_0.5_) composition at 800 °C [21,25]. Nonetheless, a clear interface can not be detected at this scale; it is thus not clear if a de-mixing of different isostructural phases takes place or, conversely, if abrupt fluctuations of the [Se]/[Te] ratio within the same phase occurs.

**Figure 3:**
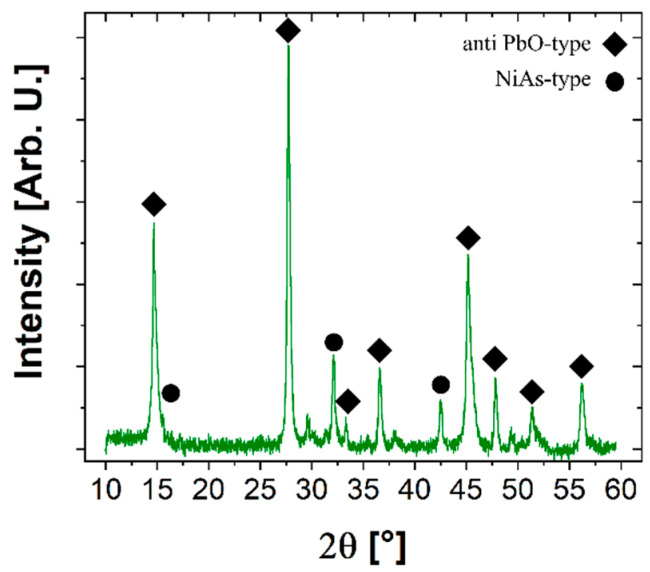
XRD pattern of the SPS pellet. The diamonds indicate the peaks of the anti-PbO-type phase and the circles indicate those of the NiAs-type phase. Non-indexed peaks do not belong to the sample but come from the sample holder. An asymmetry of the peaks of the anti-PbO-type phase is observed, indicating the coexistence of two phases: one rich in tellurium and one rich in selenium.

X-ray diffraction analysis (Figure 3) reveals that the sample is mainly constituted of the tetragonal phase (anti-PbO-type structure, space group number 129) of Fe(Se,Te) with secondary amounts of the hexagonal polymorph phase (NiAs structure, space group number 186) of FeSe. An anisotropic microstrain-like broadening of the diffraction lines is observed, increasing as the angle of the diffraction vector relative to the *c* axis tends to 0. This characteristic suggests an inhomogeneous distribution of the Se and Te, which can be ascribed to a fluctuation of the [Se]/[Te] ratio within the same phase or even a de-mixing of the tetragonal phase into isomorphic terms with different Se and Te contents, as reported also in [26,27,28]. Indeed, as also observed from the SEM images in Figure 2, there are areas where the distribution of the two elements is not uniform: from the EDS maps, regions rich in Se or Te are noticeable. In the diffraction patterns, this is reflected in an anisotropic microstrain-like broadening of the diffraction lines.

**Figure 4:**
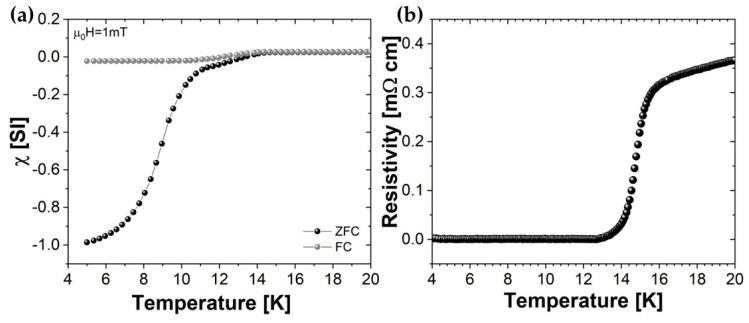
(**a**) Temperature dependence of ZFC and FC magnetic susceptibility in an applied field of 1 mT for the SPS target. (**b**) Resistivity curve as a function of the temperature for the SPS target.

Figure 4a shows the temperature dependence of the zero-field cooled (ZFC) and field-cooled (FC) dc susceptibility measured in an applied field μ_0_H = 1 mT on a slab-shaped piece of pellet obtained by SPS. We notice that a complete shielding is reached at 5 K, showing a full connectivity of the grains. The temperature at which the two curves separate is the magnetic critical temperature of the sample, which corresponds to 14 K. This value is confirmed by the resistivity measurement shown in Figure 4b, where a T_c,onset_ of about 16.5 K and a T_c0_ of about 14 K are observed on a piece of SPS pellet.

### 3.2. Film

**Figure 5:**
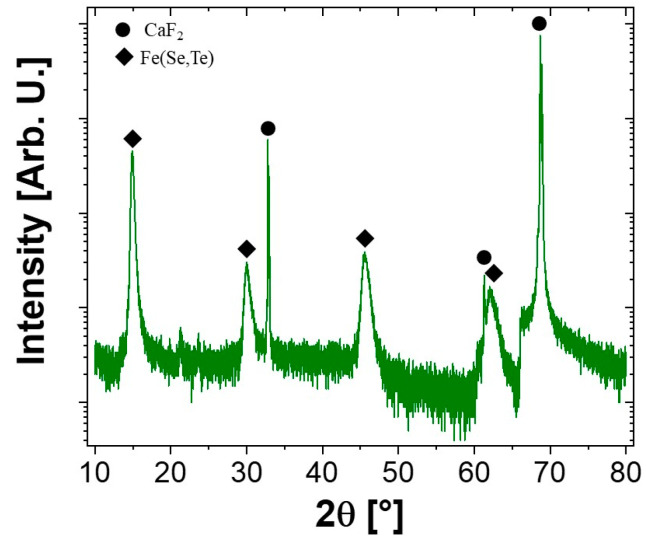
θ-2θ XRD scan of the Fe(Se,Te) thin film. (00l) peaks coming from the CaF_2_ substrate are present together with the (00l) peaks of the Fe(Se,Te) phase.

Figure 5 reports a θ-2θ scan of the Fe(Se,Te) thin film. Besides the (00l) peaks corresponding to the CaF_2_ substrate, only the (00l) reflections from the FeSe_0.5_Te_0.5_ tetragonal phase are present, indicating the excellent purity of the phase and the optimum c-axis alignment of the growth.

**Figure 6:**
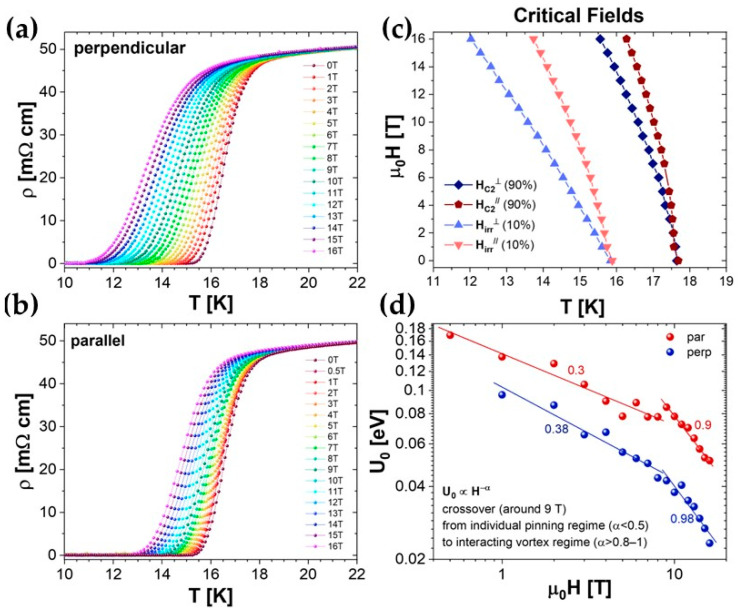
(**a**,**b**) Resistivity as a function of temperature for a thin film up to 16 T applied both perpendicular and parallel to the *ab* plane. (**c**) Upper critical field and irreversibility field evaluated as 90% and 10% of the resistivity in the normal state, respectively. (**d**) Activation energy U_0_ versus the applied magnetic field as calculated from the Arrhenius plots.

Figure 6, panels a and b, shows the resistivity curves as a function of temperature, from 0 to 16 T, with a magnetic field perpendicular (upper frame) and parallel (lower frame) to the *ab* plane. A T_c,onset_ of 18 K and a T_c,0_ of 15.5 K are observed in the zero-field spectrum. The T_c,onset_ value is in perfect agreement with the critical temperature values of films deposited using melted targets [20], while the transition is slightly less sharp [16].

From these curves, we evaluated the upper critical field H_c2_ and the irreversibility field H_irr_, taking as reference the 90% and 10% of the resistivity in the normal state (i.e., at 20 K), respectively. The trends of the two critical fields are shown in Figure 6c. The H_c2_ curves obtained for the two orientations are quite similar, and only a small anisotropy is observed: the ratio H_c2_^//ab^/H_c2_^⊥ab^ at 16 K is 1.3. H_irr_ only shows a slightly more pronounced anisotropy: the ratio H_irr_^//ab^/H_irr_^⊥ab^ at 14 K is about 1.7.

The flux motion in the mixed dissipative state is described by the thermally activated flux [29], and the resistivity—in iron-based as well as in high-temperature superconductors [30]—can be expressed by the Arrhenius relation: $\rho =\rho_{0}\left(H\right)e^{\frac{-U_{0}\left(H\right)}{k_{B}T}}$ when the currents applied for the resistivity measurements are very low. The activation energy of the vortex motion, U_0_, can be estimated by linearly fitting the Arrhenius plot (ln(*ρ*) versus 1T) of the resistivity data: in Figure 6d, U_0_ is plotted as a function of the applied magnetic field. The data are usually fitted with a low-power exponent U_0_ ∝ H^−α^, but the figure shows that a single exponent is not sufficient to fit the data over the whole range. For up to about 9 T, a weak dependence on the magnetic field is observed in both field directions, with the data being well fitted by α = 0.3 and 0.38 with the field parallel and perpendicular to the ab planes, respectively, whereas a more pronounced field dependence is observed for higher fields with α = 0.9 and 0.98 in both directions. A transition from a single-pinning regime (α < 0.5) to an interacting vortex regime (α > 0.8–1) is always observed in the literature data on U_0_ in iron-based superconductors for different field values [31,32,33].

**Figure 7:**
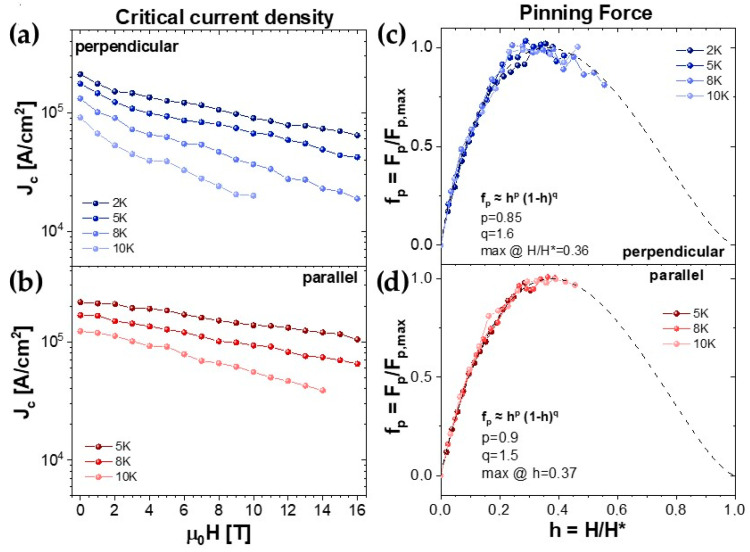
(**a**,**b**) Critical current densities up to 16 T with the field perpendicular (2, 5, 8 and 10K) and parallel (5, 8 and 10 K) to the *ab* plane. (**c**,**d**) Normalized pinning force perpendicular and parallel to the film surface, respectively.

Figure 7 shows the critical current density J_c_ of the sample measured in an applied field of up to 16 T in both the perpendicular (a) and parallel (b) configurations for different temperatures. At 5 K, J_c_ stays above 10^5^ A cm^−2^ up to 16 T when the field is parallel and up to 4 T when the field is perpendicular to the film.

From the behavior of the critical current, it is possible to extract quantitative information about the pinning force and its mechanisms. In Figure 7c,d, we show the pinning force curves normalized to their maximum value, plotted as a function of the reduced field h = H/H*, both for the parallel ad perpendicular configuration. The pinning force f_p_ = μ_0_H∙J_c_ is extracted from J_c_ measurements; the irreversibility field H* has been estimated as the value at which the J_c_^1/2^ curve is linearly extrapolated to zero: this is as an alternative to the Kramer method when dealing with point-like pinning [34]. In comparing the behavior of the pinning force in the two directions, we can see that the direction of the applied magnetic field has essentially no effect on the pinning force trend. This confirms a very weak anisotropy in these samples, both in terms of H_c2_ and pinning mechanisms.

The pinning force curves were analyzed using the Dew-Hughes model [35], which provides information on the nature and dimensionality of the pinning centers. The data were fitted by the relation f_p_ = C∙h^p^(1−h)^q^. The parameters obtained from the fit are p ≅ 0.9 and q ≅ 1.5, giving a maximum of the curves at around h_max_ = 0.37 and close to the h_max_ = 0.33 expected for point-like pinning: according to the model considered, these values are consistent with a 1D δl pinning centers (i.e., point-like centers).

## 4. Conclusions

We have used spark plasma synthesis to fabricate Fe(Se,Te) superconducting polycrystals: the pellets we produced are very dense and well connected—requirement to obtain homogeneous and highly reproducible films without particulates on the film—and show a high superconducting onset above 16 K. SPS is a promising method for synthesizing Fe(Se,Te) polycrystals for practical use as a fast densification technique suitable for the fabrication of large disk-shaped bulks in general.

The Fe(Se,Te) bulks were also used as targets to grow Fe(Se,Te) thin films through PLD. We deeply analyzed resistivity and critical current behavior of a sample deposited on CaF_2_ single crystal up to 16 T. H_c2_ is high and steep, reaching 16 T with a temperature decrease of only 1.5 K from the T_c,onset_ of 18 K, and it shows a low anisotropy. From the analysis of the activation energy of the vortex motion U_0_, we observed a transition from a single-pinning regime to an interacting vortex regime at around 9 T for both configurations of the field perpendicular and parallel to the *ab* planes. The film has a critical current density, which at 5 K is higher than 10^5^ A cm**^−^**^2^ up to 16 T when the field is parallel and up to 4 T for the when the field is perpendicular to the film. From the analysis of J_c_, we extracted information on the pinning mechanism, which resulted to be point-like pinning at all temperatures and for both directions.

## Data Availability

All the collected data were disclosed in the manuscript.
